# A Three Miles' Walk with a Penetrating Wound of the Abdomen

**Published:** 1898-04-02

**Authors:** 


### A THREE MILES' WALK WITH A PENETRATING WOUND OF THE ABDOMEN.

A series of cases reported in the *Lancet* by Dr. Zum Busch show how slight a degree of collapse may be caused even by serious penetrating wounds of the abdomen.

In the first case a man on a bicycle ran into his brother, whose bicycle had fallen, and was himself thrown, breaking the handle-bar of the machine. Both walked home a distance of three miles, and then they went to the German Hospital, as the younger one, who had been run into, had hurt his knee. When the younger brother had been attended to they were leaving the room, when the elder one—who had not up to then complained about being injured—suddenly turned pale. On being asked whether he felt any pain he pointed to a slight abrasion on the chin and said he thought his abdomen might also be slightly grazed. On examination, however, a penetrating wound of the abdomen was found with prolapse of the omentum.

In another case, a little boy suffered from a penetrating wound of the abdomen, with prolapse of the omentum, caused by falling on some broken glass, but he also walked to the hospital.

Again, a lad, aged 20 years, had been stabbed, and came to the hospital on foot; yet he had a large penetrating wound, with prolapse of omentum and of a considerable part of the small intestine.

Lastly, a woman, who had been stabbed by her husband, walked to the hospital, having a small puncture in the abdomen. On enlarging this wound, it was found to penetrate the peritoneum, the opening being filled by omentum, which was tightly gripped by it; but it was also found that the penknife had penetrated the small intestine, and had made a rather large incision into it.

All these patients, on admission to the hospital, had little idea that they were dangerously ill, and in no case was the collapse severe. From a medico-legal point of view, it is very important to recognise what a small amount of shock sometimes accompanies comparatively severe injuries.

